# Qualitative Case Study of Public Health Preparedness and Response to the Rabid Raccoon Discovered in Wise County, Virginia

**DOI:** 10.1155/2019/5734590

**Published:** 2019-04-01

**Authors:** Brian Martin, Benjamin Williamson, Vinayak K. Nahar, Karen Gruszynski, Manoj Sharma, Jason W. Johnson

**Affiliations:** ^1^Center for Animal and Human Health in Appalachia, College of Veterinary Medicine, DeBusk College of Osteopathic Medicine and School of Mathematics and Sciences, Lincoln Memorial University, Harrogate, TN, USA; ^2^Department of Dermatology, School of Medicine, University of Mississippi Medical Center, Jackson, MS, USA; ^3^Behavioral & Environmental Health, School of Public Health, Jackson State University, Jackson, MS, USA

## Abstract

Rabies is a zoonotic lyssavirus of mammals that is a major public health threat due to the high mortality rate in humans who develop clinical symptoms. In the United States and other developed countries, the main reservoirs are wildlife species. In April 2017, a raccoon tested positive for rabies in Wise County, Virginia, with a second raccoon testing positive in May. Wise County, Virginia, is one of the few counties in western Virginia that is not endemic for raccoon rabies variant virus. Due to this fact, local, state, and federal agencies worked together to prevent and control the outbreak to stop the public health theat. The purpose of this study was to understand how professionals from these various agencies viewed the response efforts to the two rabid raccoons in 2017 and to determine what could be done to improve future responses. A list of responders from the different agencies involved in the outbreak in 2017 was created. Participants were recruited via email and those who agreed to be interviewed were contacted via telephone. Participants were asked a series of 13 questions pertaining to the 2017 outbreak to understand more about the strengths and weaknesses perceived during the outbreak. Of the 11 individuals contacted, six agreed to an interview. Data were analyzed utilizing a three-step qualitative analysis process which included the steps of open coding, audit trail, and axial coding. Staff and partnerships were identified as strengths of the response while funding, community, and region were identified as weaknesses of the response. It is hoped that by identifying different strengths and weaknesses through qualitative analysis this will aid in improving future responses.

## 1. Introduction

Rabies is a zoonotic lyssavirus that is endemic in most of the world and the United States (US). It is capable of infecting and being transmitted by all mammals. In the US, the main reservoirs are wildlife species such as bats (Order Chiroptera), raccoons (*Procyon lotor)*, foxes (Family Canidae), and skunks (*Mephitis mephitis*). However, in the developing world, the primary source of infection is from dogs, due to a lower rate of canine vaccination [1]. Rabies is a major health concern for both humans and animals. In humans, rabies can cause symptoms such hydrophobia, incoordination, aggression, uncontrolled excitement, and confusion. Similar symptoms present in animals. After the appearance of symptoms, rabies is not curable, eventually resulting in death of the infected human or animal [2]. Rabies is most commonly transmitted through the bite of an infectious animal but can also be spread via brain or central nervous system tissue from an infected animal. It can be prevented in humans by the administration of postexposure prophylaxis after an exposure occurs, but before symptoms appear. Cost of the postexposure prophylaxis (PEP) is a major concern as it often exceeds $3,000 in the US [3]. In the US, rabies usually falls under the jurisdiction of public health officials and affiliated agencies as these agencies are often responsible for overseeing testing of animals, providing guidance on the administration of rabies PEP, and other activities related to rabies prevention and control.

In Virginia, the first case of raccoon rabies variant was identified in 1978. The virus gradually spread throughout the Mid-Atlantic area creating a public health burden in the area [4]. According to the Rabies Surveillance in the United States 2017 [5], Virginia had 355 animals that tested positive for rabies between January 1 and December 31, 2017 including raccoons (157), skunks (100), foxes (40), bats (20), groundhogs (2), cats (25), cattle (4), dogs (2), sheep and goats (2), and swine (1).

To control the spread of raccoon rabies variant, the United States Department of Agriculture Wildlife Service (USDA WS) has maintained the Appalachian Ridge Oral Rabies Vaccination (ORV) Zone for over 15 years [6]. The goal of the zone is to prevent the spread of rabies westward into new ecosystems. Lenowisco Health District, which consists of Wise, Lee, and Scott counties along with the city of Norton, is the major health district impacted by the vaccination zone in Virginia.

In April 2017, a raccoon tested positive for rabies in Wise County, Virginia, with a second raccoon testing positive in May [7, 8]. From the implementation of the Appalachian Ridge ORV Zone in 2002 to the racoon outbreak in 2017, there have only been a total of four bats (2002, 2008, 2013), three skunks (2002, 2009, 2011), and one dog (2003) that have tested positive for rabies in the Lenowisco Health District [9]. In response to this outbreak, public health officials advised residents to take preventative measures such as avoiding wild and stray animals and ensuring that their animal's vaccinations were up to date [8]. Additionally, the USDA WS distributed the ORV, Raboral V-RG, September through October in Tennessee, Virginia, and North Carolina as part of their Abingdon, VA project [10].

The finding of two rabid raccoons west of the zone presented a public health risk to the people in the Wise County and other communities west of the oral vaccine zone as it was an unexpected threat to the community. The purpose of this study was to evaluate the perception of professionals from various public health agencies involved in the response to the rabid raccoons in the Lenowisco Health District in 2017.

## 2. Methods

Participants were identified as having a public health related role in the prevention and control of rabies in Wise County, Virginia during the time of the discovery of the rabid raccoons. Participants were recruited via email and those who agreed were interviewed via telephone. Oral consent was received at the onset of the telephone interview and confidentiality of participants was maintained by collecting no identifiable information.

### 2.1. Interview Instrument

Participants were interviewed utilizing a standardized open-ended interview consisting of 13 questions. The first three questions established the participants' role in rabies control in Wise County, Virginia. Questions 4–6 addressed the participants' connection to the rabid raccoons. Question 7 asked the participant to rate public health preparedness in Wise County regarding rabies control while questions 8 and 9 probed what current strengths and weaknesses existed in Wise County. Questions 10 and 11 solicited opinions from the participant about what potential opportunities and barriers exist regarding rabies control in Wise County. Question 12 asked the participant if they had heard of the Center for Animal and Human Health in Appalachia (CAHA) and what role they felt it played in rabies control. The final question allowed the participants to add any additional on the topic. The questionnaire is attached as supplemental information (available here).

### 2.2. Data Analyses

Data were analyzed utilizing a three-step process [11]. The first step,* open coding, *established a thematic conceptualization around codes. The second step,* audit trail*, linked the data with themes. Finally,* axial coding* was used to put together the conceptual model. Initially, interviews were transcribed and responses were organized by question in an Excel spreadsheet. Responses were analyzed by each researcher individually for codes which were extracted to form a preliminary list of codes. A codebook was then developed by comparing individual code lists as a group so as to create a parsimonious list. This codebook was then used to code the remaining interview transcripts. Similar codes were combined into categories based on how related the codes were to each other as well as how distinct they were from other proposed categories. This process of simplification and grouping was repeated at the category stage, grouping categories into themes based on their similarities. Actual quotes were linked to each theme. Finally, the entire conceptual model was developed.

## 3. Results

Eleven participants from local, state, and federal agencies were contacted for this study. Of the 11 individuals contacted, six agreed to an interview (54.5% response rate). Interviewees had an average of 15.25 (SD: 10.69) years of experience in the area of public health or One Health and worked with the community of Wise County for an average of 10.25 (SD: 10.83) years. Participants reported working as epidemiologists, medical professionals, and health administrators. Seven categories were identified from interview results: staff, partnerships, funding, community, region, communication, and education. These categories were further placed into one of three themes:* current strengths*, which included staff and partnerships,* current barriers*, which included funding, community, and region, and finally* opportunities for improvement*, which included communication and education.

### 3.1. Current Strengths

One of the identified strength was staff quality and their ability to work with other agencies. Every participant praised the current staff and most described the current preparedness as “good” or better. In particular, all but one participant mentioned the ability of the staff to utilize partnerships with other agencies as a primary strength.

*Staff* described the quality of the individual staff members as well as staff numbers. Staff in Wise County were described as knowledgeable, proactive, experienced, and had a good physical presence within the community. Interviewee 3 stated, “I was really impressed by them, they were willing to initiate a lot of contact with the public and worked heavily with USDA Wildlife Services. They were more than willing to go above and beyond whatever was asked of them.” Interviewee 1 said “I think that the local health staff are well integrated into those communities… being present in the moment and the location where they need to be.” Regarding the staff response to the rabid raccoon, Interviewee 2 stated “They are very proactive and when an event happens it is dealt with immediately,” And “…the response, the understanding of rabies is very good, the understanding of the effects of a rabid racoon is really important and they're very respons[iv]e and they take everything very seriously.”

*Partnerships* described interactions between organizations including the Virginia Department of Health, community hospitals, universities, and even the army. Current partnerships were described as strengths but participants also mentioned the need to improve. Interviewee 4 stated, “…I think that the collaboration with veterinarians and the collaboration with animal control is good… Animal control and the health department work very closely with state labs, the Division of Consolidated Laboratory Services, that have human or other animal exposures and you know, the turnaround time and the turnaround on reporting is quite good.” Interviewee 2 likewise mentioned, “…they are one of the areas that is very inclusive with all of their partners and they have more partners than almost anyone else,” And that “…they immediately reach out and pull in people directly related and people, such as myself, who can offer assistance in either large and small ways.”

### 3.2. Current Barriers

Participants primarily described the current barriers to effective rabies control as funding, community (e.g., compliance with government regulations), and the rural region of southwest Virginia. Low community compliance and cooperation which ranged from not vaccinating pets to actively transporting animal populations across the state was also mentioned. Half of the participants mentioned the geographic and demographic barriers within the region as a weakness in terms of communicating and providing surveillance for new rabies cases.

*Funding* was primarily mentioned in terms of insufficient staff levels, as both a current weakness, and as a potential barrier to improving rabies response in the area. Low funding was implicated as the reason for decreased support of rescuing animals in neighboring counties, specifically an inability to collect and house stray cats. Interviewee 6 stated “…money is always a barrier no matter what you are talking about… they do plenty but I'm sure they could do more if they had additional staff.” It was also mentioned as a potential cause of decreased community compliance in terms of vaccinating pets. As Interviewee 4 stated, “…the cost of going to a veterinarian's office and getting a vaccine when you aren't getting it from a low-cost annual rabies clinic can cost quite a bit of money…” In terms of staff numbers, Interviewee 6 said, “The biggest weakness in my opinion is, I guess there is a lot of turnover in the emergency room staff and occasionally we get notifications of the bite sometimes 8-10 days late. And that's the exception, it's certainly not the rule. They are not always aware of the protocols and that has to do with other issues.”

*Community* described the actions of the general public acting on their own and includes topics such as compliance with vaccination and leash laws, reporting suspicious animals, and translocating animals (e.g., for maintaining population levels for hunting). Community was typically mentioned as a barrier to rabies control in terms of funding (e.g., a visit to the veterinarian's office), lack of education, or a lapse in personal accountability. Interviewee 1 claimed “…[T]here is an element of irresponsibility among pet owners… and I'm speaking of things like leaving food out, leaving water out, allowing animals to run loose, failing to vaccinate animals for rabies, all of these things are challenges that we deal with…” A culture of self-reliance was mentioned by Interviewee 3 as an impediment to compliance, saying “…they are more than willing to just take care of the problem and not call up and report that problem to the local health department and to the game department, so that testing of the carcass can occur.”

*Region* referred to the unique challenges that come with working within a rural area. Wise County's low population density was cited as a barrier to surveillance as well as communication with the public. As Interviewee 5 put it, “I certainly think that we are working together to do the best that we can, but due to the nature of the low population density and the rural nature of that area sometimes it's a little difficult to get the word out to people.” The poor socioeconomic status and sparse telecommunication infrastructure in the region also present a barrier to effective rabies control. Interviewee 3 claimed “…internet is really, really sparse down there and the economic status of many of the citizens down there doesn't allow them access to the internet…”

### 3.3. Opportunities for Improvement

Possibilities for improvement in regard to rabies control in Wise County was described primarily in terms of improving communication with the public, increasing efforts to educate the public about rabies spread and the proper methods of reporting possible rabies cases. Most participants described improvements in communication as increasing the number of outlets for releasing information about new rabies cases in order to increase the number of people receiving the news. Increasing education was mentioned by most participants in terms of increasing community compliance, especially in regard to proper vaccination of pets.

*Communication* between the health department and its partners was described as a strength in terms of being consistently prompt and inclusive. Communication with the public; however, was cited as a potential barrier due to the reasons mentioned under region. Diversification of information dissemination could improve rabies control Interviewee 2 suggested “[b]etter posting of information on social media and the routes that people use to get information other than television and newspapers.” Interviewee 3 emphasized noninternet sources, stating “Whether its leaning more heavily on public meetings or trying to get more information on to the local news as opposed to website updates and relying on the internet, which is our go to so frequently. The first thing something happens, you throw something up on your website and for this particular part of the state it's not going to work.”

*Education* refers to teaching the public about rabies prevention and surveillance. Community education was consistently seen as an opportunity to improve rabies prevention and a lack of education was mentioned as a possible cause for low community compliance. For example, Interviewee 4 stated some members of the public vaccinate animals themselves using vaccines they found at a bait store which is considered noncompliance in the state of Virginia thus illustrating a lack of knowledge about rabies laws. Interviewee 6 suggested “…coordination with the schools, educating the schools about rabies more…” as a possible avenue of improvement. In regard to improving community education, Interviewee 3 stated “I think that there may be an opportunity for that center [Center for Animal and Human Health in Appalachia] to assist with that or get the right people to the table to be able to talk about changing attitudes. Talking about the human dimension side of things, increasing communication, just increasing awareness out there.” Similarly, Interviewee 5 commented “…any group like that, especially given the locality, can help create or come up with ideas for outreach and education…”

## 4. Discussion

In this study, six of the eleven public health officials who worked on the 2017 rabid raccoon outbreak were interviewed regarding rabies prevention and control. Most interviewees believed that agencies responded to this situation in an effective and timely manner. Local health department employees, veterinarians and animal control were consistently mentioned as a strength in rabies control due to their knowledge and skill. The ability and frequency with which employees partnered with other organizations was also mentioned as a strength, but also as a potential area of improvement, particularly in areas of public outreach and education. However, while the employees were described as skilled, it was mentioned that they are short-staffed and could be more effective if they received additional funding. Funding could be used to increase staff numbers, but also to increase programs which improve public education regarding rabies, such as transmission, the importance of vaccinating pets, and the importance of working with government agencies in dealing with potentially rabid animals.

A major weakness mentioned by most interviewees was a lack of public compliance in regard to rabies control. Wise County residents were described as having an independent mentality, avoiding government involvement when they deemed it unnecessary (e.g., disposing of a rabid animal). Residents were noted to sometimes leave food and water out for wild animals, increasing the chances of contracting rabies by either themselves or their pets. There is also the likelihood of residents transporting raccoons across the state in an effort to maintain/increase population levels. These findings suggest there is a general lack of education regarding rabies among the residents of Wise County and underline the importance of increasing efforts to educate the public about the dangers of rabies and handling wild animals and the importance of vaccinating pets.

Public compliance is of utmost importance when attempting to control rabies for many reasons. Rabies is a resilient virus which has successfully established itself in many mammalian species. In 2018, Ma et al. [6] found that 2/3 of reported rabies cases in the US were not variant typed, increasing the risk of a rabies case going unidentified, and possibly missing the spread of a known variant into a new environment. Unreported cases (e.g., when individuals do not report animals behaving strangely) naturally increase the risk of allowing a rabies case to go unidentified.

Communication with the public was mentioned as an area of difficulty, primarily due to the rural nature of Wise County. Internet access was described as being sparse and not wholly reliable as a means of disseminating knowledge to the average citizen. Barring improvements to telecommunications infrastructure, more traditional means of reaching and educating the public may be necessary and could be a task undertaken by Lincoln Memorial University's Center for Animal and Human Health in Appalachia (CAHA). Half of the participants indicated a potential role for CAHA in increasing the presence of veterinarians and medical professionals familiar with rabies virus and other zoonoses. CAHA was viewed as a way to increase public knowledge through either educational campaigns or outreach such as town meetings regarding vaccinations and animal health.

From a government standpoint, continued use of ORV remains an effective method of controlling rabies. However, it is an object of concern that the two raccoons were found west of the Appalachian ORV, which brings up questions on how the raccoons arrived there. In neighboring West Virginia, Plants et al. [12] found significant decreases in the number of rabies-infected racoons from 2000-2015 due to heavy ORV utilization. However, they found that ORV was less effective against nonraccoon hosts, suggesting possible viral spread to other animals such as pets and livestock and emphasizing the need for regular vaccinations of animals in contact with humans. In a Canadian study on rabies containment in raccoon populations, Stevenson et al. [13] found that rabies could be eliminated from raccoon populations through oral vaccine baiting tailored to raccoon environments and following up on public reports. However, they found that in the provinces of Quebec, New Brunswick, and Ontario that rabies returned to the raccoon population after a period as little as four years, underscoring the importance of continued vigilance in surveillance and baiting. Freuling et al. [14] described the necessity of increased funding needs for total rabies elimination in their study of the eradication of rabies from fox populations in western and central Europe. They noted diminishing returns to oral vaccination efforts which necessitated a determined effort to reach total elimination, rather than simply diminishing rates of occurrence.

## 5. Conclusion

Overall, the public health officials in Wise County, Virginia thought that the response to the 2017 rabies outbreak in raccoons was effective. Strengths included experienced staff and partnerships with local and national agencies. Funding, compliance, and communication were identified as areas that could be improved upon in the future. These findings are not only of importance to Wise County, Virginia, but could be of value to other rural, raccoon variant endemic areas in the Eastern US.

## Data Availability

The data used to support the findings of this study are available from the corresponding author upon request.

## Supplementary Materials

Supplementary Materials Interview instrument measuring public health preparedness and response to the rabid raccoon.
