# Supplementary material for: Qualitative Case Study of Public Health Preparedness and Response to the Rabid Raccoon Discovered in Wise County, Virginia
Source: J Vet Med. 2019 Apr 1;2019:5734590. doi: 10.1155/2019/5734590 (PMC6463573; doi:10.1155/2019/5734590)
Supplement: Supplementary Materials — Interview instrument measuring public health preparedness and response to the rabid raccoon. [file 5734590.f1.docx]

**Qualitative Case Study of Public Health Preparedness and Response to the Rabid Raccoon Discovered In Wise County, Virginia**

**Interview Instrument**

*Salutation script:* Hello. I am ____________. I am a student working with the Center for Animal and Human Health in Appalachia at Lincoln Memorial University working under the supervision of Dr. Karen Gruszynski, Assistant Professor of College Veterinary Medicine at the Lincoln Memorial University (LMU). You have been identified as a public health professional associated in dealing with the recent rabid raccoon discovered in Wise County. We will be asking you a set of 13 questions to describe public health preparedness and response as whole in this event and in such future events. Completing the interview is voluntary and will not change the relationship with LMU if not completed. The interview will be recorded and destroyed 3 years after the completion of the study as mandated by federal law. This project has received LMU Institutional Review Board approval. Your responses will help in assessing strengths and weaknesses learned in the context of rabies in Wise County.

1. How long have you worked in the area of public health or One Health?
2. How long have you worked in Wise County?
3. Please describe the nature of your job.
4. When did you first hear of the rabid raccoon in Wise County?
5. What were your initial steps in regard to the rabid raccoon in Wise County?
6. Did you collaborate with anyone? If so, with whom and how?
7. How would you rate the public health preparedness with regard to dealing with rabies in Wise County?
8. What are the current strengths in regard to dealing with rabies in Wise County?
9. What are the current weaknesses in regard to dealing with rabies in Wise County?
10. What are some potential future opportunities that you see in regard to improving prevention and control related to rabies in Wise County?
11. What are some any potential future barriers you perceive in regard to prevention and control of rabies in Wise County?
12. Have you heard about the Center for Animal and Human Health in Appalachia or CAHA at Lincoln Memorial University in TN? If so what role do you think CAHA can play in regard to rabies?
13. Anything else you would like to add?

Thank you very much for your time. Do you need summative results of these interviews? If yes, then please provide your e–mail.
